# Efficacy and safety of MK-7243: a grass allergy sublingual immunotherapy tablet evaluated in Canadian adults and children

**DOI:** 10.1186/1710-1492-10-S2-A16

**Published:** 2014-12-18

**Authors:** Jacques Hébert, Michael Blaiss, Susan Waserman, Harold Kim, Peter Creticos, Jennifer Maloney, Amarjot Kaur, Harold S Nelson, Hendrik Nolte

**Affiliations:** 1 Centre de Recherche Appliquée en Allergie de Québec, Québec City, QC, Canada; 2 Departments of Medicine and Pediatrics, University of Tennessee Health Science Center, Memphis, TN, USA; 3 Department of Medicine, McMaster University, Hamilton, ON, Canada; 4 Department of Medicine, Johns Hopkins University School of Medicine, Baltimore, MD, USA; 5 Merck & Co., Inc., Whitehouse Station, NJ, USA; 6 Departments of Medicine and Pediatrics, National Jewish Health, Denver, CO, USA

## Background

The effect of MK-7243 (2800 BAU/75,000 SQ [~15 µg of *Phleum pratense p* 5], Merck/ALK-Abelló), a sublingual Timothy grass immunotherapy tablet, has been evaluated in several randomized, placebo-controlled, double-blind trials; three of these trials were conducted in adults and children in North America (the United States and Canada) who have allergic rhinitis with or without conjunctivitis (AR/C). We conducted a post-hoc analysis to investigate the effect of MK-7243 in Canadian subpopulations.

## Methods

Data from Canadian subjects from the three trials were used in this investigation: P05238 (adults ≥18 y; pollen season: 2009); P05239 (children 5−<18 y; pollen season=2009); and P08067 (adults ≥65 y and children 5−<18 y; pollen season: 2012). Trial data from the same grass pollen seasons (GPS) were pooled. Subjects received once-daily MK-7243 or placebo starting ≥12 wk before and continuing throughout the GPS, for a mean total of ≥23 wk. The therapeutic effect of MK-7243 was evaluated for rhinoconjunctivitis symptoms and symptomatic medication use, measured as a total combined score (TCS=daily-symptom score [DSS; max=18]+daily-medication score [DMS; max=36]) averaged over the entire GPS. Safety was assessed by monitoring adverse events (AEs).

## Results

Canadian subjects taking MK-7243 (n=42) in the pooled adult-pediatric 2009 trials showed a 38% mean TCS reduction versus placebo (n=54; −2.06 difference [95% CI: −3.72, −0.39]; 3.32 vs. 5.37). Canadian subjects taking MK-7243 (n=122) in the adult-pediatric 2012 trial showed a 33% mean TCS reduction relative to placebo (n=122; −1.62 difference [95% CI: −2.54, −0.71]; 3.34 vs. 4.96). Approximately 90% of treatment-related AEs were mild or moderate in severity. No serious or life-threatening treatment-related AEs occurred.

## Conclusions

MK-7243 Timothy grass sublingual tablet significantly improved AR/C induced by Timothy grass pollen in Canadian adults and children 5 y and older. Similar efficacy and safety results were obtained for the overall populations of the three trials.

## Trial registration

ClinicalTrials.gov Identifiers: NCT00562159; NCT00550550; NCT01385371

